# Prevalence and mitigation of aflatoxins in Kenya (1960-to date)

**DOI:** 10.3920/WMJ2018.2362

**Published:** 2018-09-11

**Authors:** C.K. Mutegi, P.J. Cotty, R. Bandyopadhyay

**Affiliations:** 1 International Institute of Tropical Agriculture, IITA, c/o ILRI, P.O. Box 30709, Nairobi 00100, Kenya; 2 United States Department of Agriculture, Agricultural Research Service, 416 West Congress Street, Tucson, AZ 85701, USA; 3 International Institute of Tropical Agriculture, IITA, PMB 5320, Ibadan, Nigeria

**Keywords:** aflatoxicosis, liver cancer, economic drivers, knowledge gaps

## Abstract

Aflatoxins are highly toxic metabolites of several *Aspergillus* species widely distributed throughout the environment. These toxins have adverse effects on humans and livestock at a few micrograms per kilogram (μg/kg) concentrations. Strict regulations on the concentrations of aflatoxins allowed in food and feed exist in many nations in the developing world. Loopholes in implementing regulations result in the consumption of dangerous concentrations of aflatoxins. In Kenya, where ‘farm-to-mouth’ crops become severely contaminated, solutions to the aflatoxins problem are needed. Across the decades, aflatoxins have repeatedly caused loss of human and animal life. A prerequisite to developing viable solutions for managing aflatoxins is understanding the geographical distribution and severity of food and feed contamination, and the impact on lives. This review discusses the scope of the aflatoxins problem and management efforts by various players in Kenya. Economic drivers likely to influence the choice of aflatoxins management options include historical adverse health effects on humans and animals, cost of intervention for mitigation of aflatoxins, knowledge about aflatoxins and their impact, incentives for aflatoxins safe food and intended scope of use of interventions. It also highlights knowledge gaps that can direct future management efforts. These include: sparse documented information on human exposure; few robust tools to accurately measure economic impact in widely unstructured value chains; lack of long-term impact studies on benefits of aflatoxins mitigation; inadequate sampling mechanisms in smallholder farms and grain holding stores/containers; overlooking social learning networks in technology uptake and lack of in-depth studies on an array of aflatoxins control measures followed in households. The review proposes improved linkages between agriculture, nutrition and health sectors to address aflatoxins contamination better. Sustained public awareness at all levels, capacity building and aflatoxins related policies are necessary to support management initiatives.

## Introduction

Aflatoxins are natural metabolites of several *Aspergillus* species. The aflatoxin producing species within section *Flavi* most frequently associated with crop commodities in Africa is the L-strain morphotype of *Aspergillus flavus*, with lesser occurrences of *Aspergillus parasiticus*, *Aspergillus tamarii*, and isolates of S-morphology (Probst *et al.,* 2014). Species less frequently associated with crops include *Aspergillus nomius, Aspergillus pseudotamarii* and *Aspergillus bombycis* (Peterson *et al.*, 2001). Aflatoxin producing fungi have a broad host range including several cereal crops, oil seed crops, legumes, tree nuts and many other domestic and non-cultivated plants (Klich, 2007). The potent effects of aflatoxins on humans and adverse effects on trade have triggered the extensive study of aflatoxins globally. Aflatoxin B_1_, the most common of at least 14 different forms (Boutrif, 1998), is classified by the International Agency for Research on Cancer (IARC) as a group 1 carcinogen (Ostry *et al.,* 2017), because of its ability to bind with DNA and cause hepatocellular carcinoma in both animals and humans. Crops grown under tropic and sub-tropic conditions have the greatest frequency of unacceptable aflatoxins content. This region is characterised by a tropical climate with periodic droughts and temperatures frequently exceeding 28 °C and high humidity, with terrains undergoing the negative impacts of climate change (Cotty and Jaime-Garcia, 2007; Negash, 2018; Ongoma, 2013). Contamination of produce with aflatoxins and its impact in Kenya has been dire and is evidenced by the periodic reported incidences of acute aflatoxicosis as well as alarming levels of chronic exposure amongst most of the Kenyan population (Gong *et al.*, 2012). As a result, aflatoxins issues in Kenya have received a great deal of attention from the academia, policy makers, farmers, processors, the international community and food aid agencies, among others.

In the academia, peer-reviewed articles are considered as the gold standard for seeking authentic information. Old literature and various sorts of information from unorthodox sources are generally ignored despite sometimes providing unusual insights on aflatoxins issues, such as socio-cultural aspects of the problem. We reviewed contamination of aflatoxins in Kenya using published literature, grey literature and media sources. The purpose of the review is to consolidate existing information about aflatoxins contamination and exposure in Kenya, taking into account the historical perspective; consolidate information on efforts on aflatoxins mitigation in Kenya by discussing institutional roles; highlight economic drivers likely to influence the scaling of mitigation measures; and identify knowledge gaps for guiding future aflatoxins management efforts.

## Prevalence of aflatoxins in Kenya

### Aflatoxins in food and feed

Little work on the prevalence of aflatoxins in Kenya was published before the 21^st^ century. Early studies (Peers and Linsell, 1973) found high incidence of contamination with aflatoxins in the majority (93%) of the main meals and local brew from households in the Murang’a District of Kenya. The meals composed of maize, millet, sorghum, pigeon peas and yam components. Several studies undertaken in the 21^st^ century have consistently shown aflatoxins in a variety of foodstuffs and from various regions in Kenya (Daniel *et al.*, 2011; Gachomo *et al.*, 2004; Keter *et al.,* 2017; Lewis *et al.*, 2005; Menza *et al.*, 2015; Mutegi *et al.*, 2009, 2010, 2013; Mutiga *et al.*, 2014, 2015; Mwihia *et al.*, 2008; Sirma *et al.,* 2016). Of interest from several of these publications are the alarmingly high proportions (Table 1) of food commodities that surpass the Kenyan regulatory threshold of 10 μg/kg set for total aflatoxins and 5 μg/kg set for aflatoxin B_1_ content (KEBS, 2018a). Notably, the very high levels were recorded in Kenya’s main staple starch, maize, peanuts and in animal feed. These statistics, coupled with the regular consumption of sizeable portions of maize products across diverse age groups (Kang’ethe *et al.,* 2017), provide insights into the high chronic aflatoxins exposure rates in the country, of about 67% of the population (Githang’a and Awuor, 2016). Further, the wide ranges of aflatoxins whose upper limits in many instances stretch to four-digit μg/kg values (Table 1) have on many occasions resulted in death.

**Table 1:** Selected data on aflatoxins prevalence in Kenya (1960-date) from published sources.

Subject Origin	Range ()^a^	Above threshold (%)^b^	n	Reference
Maize products (μg/kg)				
Makueni, Kitui	LOD-48,000 (9.1 Gm)	35^c^	716	Daniel *et al.,* 2011
Nairobi (Korogocho; Dagoretti)^g^	0-88.83 (6.7); 0-20 (2.97)	16	99; 87	Kiarie *et al.,* 2016
Kitui, Makueni, Machakos, Thika	1.0-46,400 (20.53)	55 ^c^	342	Lewis *et al.*, 2005
Eastern, Nyanza	0.01-9,091.8 (46.9)	50.3	789	Mahuku, 2018 unpublished data
Western Kenya	LOD-710	15	985	Mutiga *et al.,* 2015
Upper and Lower eastern	LOD-4,839	39	1,500	Mutiga *et al.,* 2014
Makueni	0.0-13,000	35.5 ^c^	104	Mwihia *et al.,* 2008
Nairobi	0.11-4,593	83	144	Okoth and Kola, 2012
Kwale, Isiolo, Tharaka Nithi, Kisii, Bungoma	<1.0-1,137	26 ^d^	497	Sirma *et al.,* 2016
Peanut products (μg/kg)				
Busia, Kisii	0.1-591.1	48.8 ^d^	204	Menza *et al.,* 2015
Nyanza, Western, Nairobi	LOD-32,328	37	1,161	Mutegi *et al.,* 2013
Busia, Homabay	0.0-7,525	7.5 ^c^	769	Mutegi *et al.,* 2009
Nairobi, Nyanza	LOD-2,377	43	82	Ndung’u *et al.,* 2013
Eldoret and Kericho towns	0.0-2,345	–	228	Nyirahakizimana *et al.,* 2013
Sorghum (μg/kg)				
Nairobi (Korogocho; Dagoretti )^g^	0.2-194.41 (8.07); 0.1-14.47 (2.59)	11	53; 36	Kiarie *et al.*, 2016
Kwale, Isiolo, Tharaka Nithi, Kisii, Bungoma	<1.0-91.7	11 ^d^	164	Sirma *et al.,* 2016
Millet (μg/kg)				
Kwale, Isiolo, Tharaka Nithi, Kisii, Bungoma	<1.0-1,658.2	10 ^d^	205	Sirma *et al.,* 2016
Medicinal herbs (μg/kg)				
Eldoret and Mombasa towns	<0.25-24	–	100	Keter *et al.*, 2017
Milk products (ng/kg)				
Eldoret, Machakos, Nyeri, Machakos, Nakuru, Nairobi	5.8-600	20 ^e^	613	Kang’ethe and Lang’a, 2009
Makueni	1.4-152.7 (0.83)	22.2 (detected)	18	Kang’ethe *et al.,* 2017
Nandi	0.5-0.8 (0.06)	9.5 (detected)	21	Kang’ethe *et al.,* 2017
Nairobi (Korogocho; Dagoretti) ^g^	0.002-2.56 (0.132); 0.007-0.64 (0.093)	63 ^e^	76; 52	Kiarie *et al.*, 2016
Nairobi	LOD-1,675	55 ^e^	190	Kirino *et al.,* 2016
Bomet	LOD-2.93	43.8 ^e^	156	Langat *et al.,* 2016
Kwale, Isiolo, Tharaka Nithi, Kisii, Bungoma	<2-6,999 (3.2 Gm)	10.4 ^e^	512 (farmers)	Senerwa *et al.,* 2016
Animal feed products (μg/kg)				
Eldoret, Machakos, Nyeri, Machakos, Nakuru, Nairobi	–	67 ^d^	830	Kang’ethe and Lang’a, 2009
Nairobi	5.13-1,123	95	72	Okoth and Kola, 2013
Kwale, Isiolo, Tharaka Nithi, Kisii, Bungoma	<1.0-4,682 (9.8 Gm)	61.8 ^d^	102 (feed manufacturers)	Senerwa *et al.,* 2016
Kwale, Isiolo, Tharaka Nithi, Kisii, Bungoma	<1.0-1,198 (25.6 Gm)	90.3 ^d^	31 (feed retailers)	Senerwa *et al.,* 2016
Human exposure (pg/mg) AFB_1_-lysine adduct level				
Various	0.05-0.417 (AFB-gual)	12.6 (detected)	830	Autrup *et al.,* 1987
Tharaka Nithi and Meru Counties	4.18-10.46 (7.82)	100 (detected) ^f^	884	Leroy *et al.,* 2015
Nyanza, Coast, eastern, Rift Valley	LOD-211 (2.01 Gm)	78 (detected)	597	Yard *et al.,* 2013

a Mean values in brackets; in some instances the authors did not present mean values and in other, Arithmetic mean was not differentiated from geometric mean (Gm).

b Percent beyond Kenyan regulatory threshold (10 μg/kg).

c Percentage based on the then Kenyan regulatory threshold of 20 μg/kg.

d Percentage samples is based on the KEBS regulatory threshold for aflatoxin B_1_ (5 μg/kg).

e Percentage based on a threshold of 0.05 μg/kg.

f Sample size comprised of women.

g Korogocho is a slum neighbourhood of Nairobi and Dagoretti is one of the eight divisions of Nairobi; aflatoxin data are provided for both areas, respectively.

Various government departments are a source of prevalence data through their monitoring and surveillance programs. Reports from the National Cereals and Produce Board (NCPB) show varying contamination levels across the country, with high levels reported from eastern Kenya (unpublished data by National Cereals and Produce Board (NCPB), 2012) and undetectable levels in some other areas (NCPB, 2016, Laboratory analysis report no. NCPB MA 1/B/ANALAB (Quality control laboratory of the National Cereals and Produce Board, Nairobi grain Silos, unpublished data). Such contamination has been attributed to growing maize in ecologically predisposed regions of Kenya (Azziz-Baumgartner *et al.,* 2005; Daniel *et al.*, 2011; Lewis *et al.*, 2005; Mutiga *et al.*, 2014; Mwihia *et al.*, 2008), mono-cropping, growing produce on smallsized farms, sub-humid agro-ecologies, broken kernels, poor ventilation (Mutiga *et al.*, 2014), high moisture content in harvested grain, drying of grain directly on soil surface (bare ground), insect damage, storage of grain in propylene bags, poor aeration of stored grain, informal marketing structures (Mutegi *et al.*, 2009, 2013; Nyirahakizimana *et al.*, 2013; Wagacha and Muthomi, 2008), lack of collective action (Mutegi *et al.*, 2007) and poverty (Leroy *et al.*, 2015). High incidences of aflatoxins in maize have in fact been reported from multi-year in-country studies (Daniel *et al.,* 2011; G. Mahuku, unpublished data; Okoth and Kola, 2012; Xu *et al.,* 2018), confirming that such incidences are not one-off occurrences.

There are large quantities of fungi in *Aspergillus* section *Flavi* associated with both maize and peanuts produced in Kenya, and there is a positive correlation between the frequency of aflatoxin producing fungi and levels of aflatoxins (Mutegi *et al.*, 2009; Muthomi *et al.*, 2009; Probst *et al.,* 2007; Wagacha *et al.,* 2013). Members of *Aspergillus* section *Flavi* with both L-strain and S-strain morphologies are present on crops produced in Kenya, and members of each may produce significant quantities of aflatoxins (Okoth *et al.*, 2012, 2018; Probst *et al.*, 2007, 2010). However, aetiology of the high concentrations of aflatoxins in maize associated with human aflatoxicoses has been attributed to novel S-morphology aflatoxin producers initially discovered in contaminated maize from the lethal aflatoxicosis outbreak in Kenya during 2004 to 2006 (Probst, *et al.*, 2007, 2012). *Aspergillus* community structure has an important influence on the extent to which maize becomes contaminated with aflatoxins (Probst *et al.*, 2010). In highly contaminated maize from low elevation areas of Kenya, there is a high prevalence of the novel S-morphology aflatoxin producers which were a previously unknown complex of aflatoxin producing fungi. Members of this phylogenetically distinct complex of highly toxic aflatoxin producers have in common a complex specific indel inthe *cypA*/*norB* region of the aflatoxins biosynthesis gene cluster (Probst *et al.,* 2012, 2014). This indel prevents this group of fungi from producing G aflatoxins. However, the group does produce very high concentrations of the most toxic aflatoxin, aflatoxin B_1_. The complex of highly toxic S morphology fungi occurred in frequencies sometimes exceeding 90% of the *Aspergillus* section *Flavi* in maize grain samples containing >1,000 μg/kg and consumed by people who died from aflatoxicosis (Probst *et al.*, 2007). Fungi belonging to this new S morphology complex are most prevalent in the low elevations where most acute aflatoxicosis cases occurred and are less frequently encountered in other regions (Probst *et al.*, 2010).

High aflatoxin M_1_ levels have been found in milk from various urban and peri-urban areas of Kenya including in pasteurised and ultra-heat-treated milk. Langat *et al.* (2016) found that almost half (43.8%) of all samples and mainly unprocessed milk had levels above 0.05 μg/kg aflatoxin M_1_ in milk, while Kang’ethe and Lang’a (2009) established that at least one in every five milk samples from urban dairy farmers and market outlets exceeded this level of 0.05 μg/kg aflatoxin M_1_ content. Summary data (Table 1) not only reveals high levels of aflatoxin M_1_ in milk that is traded informally (Kirino *et al.,* 2016), but also shows varied contamination in milk produced by livestock from various agro-ecologies (Senerwa *et al.,* 2016). The high contamination levels in milk and other animal products could be explained by the heavily contaminated animal feed found in many parts of the country (Gathumbi, 1993; Kang’ethe and Lang’a, 2009; Okoth and Kola, 2012; Rodrigues *et al.*, 2011; Senerwa *et al.,* 2016; Table 1). Due to the lack of knowledge and options for disposal of contaminated grain at the household level, contaminated grain is eventually fed to domestic animals (Kiama *et al.,* 2016; Mutegi *et al.,* 2007). Also, grain traders may exploit trade loopholes by diverting aflatoxin contaminated grain into animal feed manufacturing enterprises (East African Community, 2018a).

### Aflatoxins and health

That dietary staples contaminated with aflatoxins could be an aetiological factor in liver cancer was first suggested by Le Breton *et al.* (1962). Linsell (1967) used biopsy material to establish that the Kamba tribe of Kenya had a frequency of liver cancer that was approximately twice that of the Kikuyu ethnic community. Not surprisingly, the majority of the acute aflatoxicosis incidences have been reported in eastern Kenya where the Kamba community resides (Lewis *et al.*, 2005; Ngindu *et al.*, 1982). Peers and Linsell (1973) also established a statistically significant association between aflatoxin levels and liver cancer incidence in residents of the Murang’a District. It has been suggested that hepatitis B virus may be a co-factor in the aetiology of liver cancer (Bagshawe *et al.*, 1975). The role of a genetic factor in the induction of liver cancer by AFB_1_ was also alluded to (Autrup *et al.*, 1987).

The first recorded acute human aflatoxicosis outbreak in Kenya occurred in 1981 involving 12 fatalities (Ngindu *et al.*, 1982), while the second occurred in the 2004-2005 cropping season (Lewis *et al.*, 2005) with both incidents occurring in lower eastern Kenya. High aflatoxins concentrations in food, serum aflatoxin B_1_-lysine adduct concentrations and positive hepatitis B surface antigen titres have been identified as risk indicators for aflatoxicosis and hepatocellular carcinoma in Kenyan populations (Azziz-Baumgartner *et al.*, 2005; Wambui *et al.*, 2016a).

Exposure to aflatoxins in Kenya begins from infancy since breast milk, the first source of nourishment, of a high proportion of mothers tested positive for aflatoxin M_1_ (Kang’ethe *et al.* 2017). The proportion ranged from 56.7% in Nandi County to 86.7% in Makueni County. Kang’ethe *et al.* (2017) further demonstrated the high exposure in aflatoxin M_1_ levels found in urine samples from children under 2.5 years (mean of 1.182 μg/kg and 0.857 μg/kg aflatoxin M_1_ in Makueni and Nandi counties, respectively). Even though direct link between exposure and malnutrition was not investigated in the study, both counties had stunted and severely stunted children above the national averages of 26% and 11%, respectively (KNBS and ICF Macro, 2010). The role of contamination from diets and exposure on the nutritional status of children needs to be investigated further. Exposure to aflatoxins has been linked with malnutrition among children from various parts of the country (Kang’ethe *et al.,* 2017; Kiarie *et al.,* 2016; Okoth and Ohingo, 2004). High exposure levels in human populations in Eastern and Coastal provinces of Kenya have also been observed by Yard *et al.* (2013) while alarming statistics of 100% exposure in pregnant and lactating mothers from Eastern Kenya were established by Leroy *et al.* (2015). Such high exposure levels that rank higher than other countries within sub-Saharan Africa (Xu *et al.,* 2018) can be partly explained by the elevated contamination levels in foodstuffs discussed earlier.

Aflatoxicosis incidences in animals have also been reported in published literature, albeit far and apart (Mbugua and Etale, 1987). Grey literature reports on aflatoxicosis in animals have been documented by the public health department, alongside human aflatoxicosis incidences (Table 2).

**Table 2:** Reported aflatoxicosis cases from public health records in Kenya (Ministry of Public Health and Sanitation, 2012).

Year of occurrence	Nature of occurrence
1960	16,000 ducklings from white settler farms in Rift Valley Province die from aflatoxin contaminated groundnut feed
1977	Large numbers of dogs and poultry die in Nairobi, Mombasa and Eldoret from contaminated produce due to poor storage
1984-1985	Large numbers of poultry die after being fed imported contaminated maize
1998	Three humans from Meru North die from eating aflatoxin contaminated maize
2001	26 humans from Maua suffer severe liver damage from aflatoxin contaminated maize
2003	Six humans die from contaminated maize in Thika
2004	331 humans reported with various levels of aflatoxicosis after consuming aflatoxin contaminated grain that resulted to 125 deaths in Eastern/Central Machakos, Kitui and Makueni areas
2006	Ten human deaths in Mutomo, and nine in Makueni linked to consumption of aflatoxin contaminated maize
2007	Two human deaths reported in Makindu
2008	Two human deaths reported in Kibwezi. Three persons hospitalized in Mutomo
2010	Unconfirmed cases of dogs dying in Nairobi

## Aflatoxins in the Kenya press

**Figure 1:**
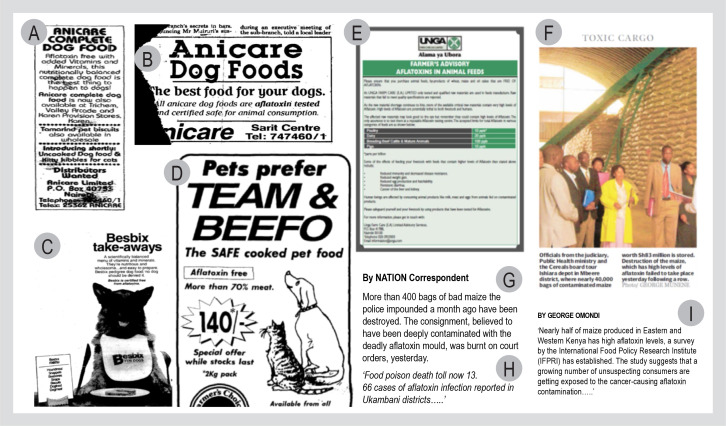
Examples of mentions of aflatoxins in print media: (A) 1985; (B) 1989; (C) 1993; (D) 1998; (E) 2008; (F) 2009; (G) 2004; (H) 2005; and (I) 2011. Early mentions in print media were related to pet food and animal feed.

**Figure 2:**
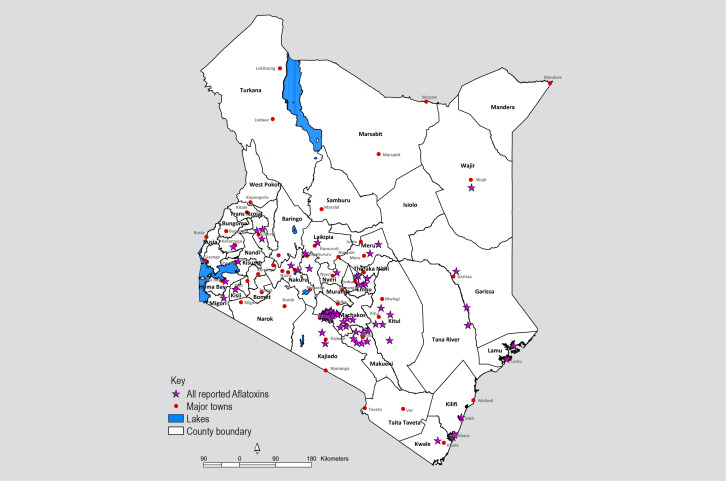
Geographical spread of media reports on aflatoxins across the country between 1960 to date.

**Figure 3:**
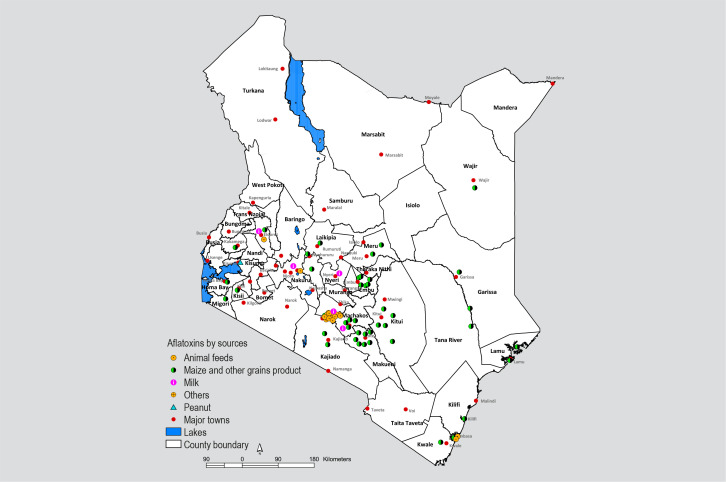
Geographical spread of media reports on aflatoxins in various food and feed commodities in the country between 1960 to date.

Up to the 21^st^ century, published data about aflatoxins was scanty and media filled a void in reporting fatalities and contamination particularly in domestic animals and feed (Figure 1A, B, C, D and E). The 21^st^ century witnessed a surge in reporting contamination in food products in peer-reviewed literature discussed previously and through media reports (Figure 1F, G, H, and I; Table 3). Table 3 lists media mentions and their thrust that depict an increase in reporting about aflatoxins over the years. Albeit regular aflatoxins contamination mentions in produce from Eastern and Nairobi provinces, the spread of reporting in other regions allude to a nationwide problem (Figure 2) rather than regional, and across various food commodities (Figure 3).

**Table 3:** A chronological listing of summarised accounts on aflatoxins related reporting from Kenyan media houses.^1^

Year	Mentions	Origin	Key accounts
1960-1969	3	Nation Media Group	Concern by experts grows on possible high aflatoxin levels in peanuts destined for the developed world.
			Highlights on the peanut management through grading, sizing and separation of contaminated nuts.
1970-1979	19	Daily Nation	Alert on the death of pigs in America in 1954 fed on contaminated maize.
			Several deaths of domesticated dogs, quails and ducks in Kenya after consuming contaminated processed food; these led to government chemists requesting manufacturers to withdraw their products from the market; and Kenya Society for the Protection and Care of Animals warns their members against giving their pets branded feed. Led to one manufacturer halting the sale of six of their brands for dog feed.
			Kenya, Ivory Coast and Swaziland reported to have the highest rate of liver cancer in the continent, which was attributed to aflatoxins contaminated grain.
			Role of storage in aflatoxins contamination highlighted.
			Several calls by the public to probe human and animal feed manufacturing companies for aflatoxins contamination in their products.
1980-1989	24	Daily Nation	Informs of availability of an aflatoxin testing kit in the Agricultural Society of Kenya (ASK) shows.
			Major concerns on human health from aflatoxins contamination were raised on several occasions.
			The first major aflatoxicosis fatality in Kenya reported; 6 members of one family (a man, his wife and four children) were among the dead.
			Major food grain losses due to contamination were reported.
			Government orders a shipload of imported maize grain to be thrown into the sea due to high levels of aflatoxins recovered in the grain.
			Contamination in other foods, such as *chan’gaa*^2^ was reported.
			Further reports on death of dogs from eating contaminated feed continue.
			Role of storage in aflatoxins receives more attention.
			In 1985, a committee of stakeholders in the animal/livestock sector was established to set up standards for animal feed.
1990-1999	9	Daily Nation	Albeit fewer, majority of the reports were on aflatoxin contamination in food and human health.
			Many commercial maize flour samples were contaminated with zearalenone, ochratoxin and aflatoxins beyond the regulatory threshold.
			National Cereals Produce Board ordered by government to destroy tons of imported maize stock contaminated with aflatoxins.
2000-2009	53		Most reports skewed towards human poisoning and food contamination.
			The worst aflatoxicosis incidence was reported between the 2004-2005 cropping season; another one was reported in 2008.
			Public health officials crack down and destroy contaminated maize in Kajiado and Laikipia (Rift Valley), Kilifi and Bura (Coast), Mbeere and Embu (Eastern) and Wajir (North Eastern Province), most of the maize meant for distribution in schools.
			Issues related to aflatoxins contamination in maize raised by the Members of Parliament.
			Several reports made on maize that was contaminated and went missing.
			150 dogs reported dead in Mombasa after eating contaminated feed.
			High levels of contamination in milk and livestock feed highlighted from urban and peri-urban areas.
			Premature harvesting of maize highlighted as a contributing factor to aflatoxins contamination.
			Focus begins to shift to intervention discussions.
			Government introduces the warehouse receipting system.
			FAO commits to work with government to introduce measures to arrest further contamination.
2010-to date	>100		Reporting on aflatoxins issues increases drastically.
			Surveillance measures were heightened with maize produce from Eastern, Western, Central, and Nairobi Provinces being confiscated.
			28 tons of a supplementary porridge flour were recalled due to high levels of aflatoxins contamination. The contaminated maize had been distributed to thousands of school-going children, in Eastern, Central, Coast and North Eastern provinces.
			Media highlights that 40% of maize from Eastern and Western Kenya could not meet the Kenya Bureau of Standard (KEBS) aflatoxins regulatory threshold.
			Government through the Ministries of Agriculture and Health announce introduction of various post-harvest measures for aflatoxins mitigation, including awareness raising.
			COMESA commits to providing aflatoxins contaminated food alerts for traders in the region.

1 The authors acknowledge that several mentions could have been left out as they depended on the archives of the major media houses consulted, many of which did not have computerized documenting systems by the time the reports were filed, making it difficult to access all relevant information.

2 A local alcoholic drink made by spontaneous fermentation and distillation process usually of maize or sorghum.

Data from published sources expose information gaps over long periods even though no unique factor was present during the gap periods to imply lack of contamination. In fact, over the years, environmental factors, climate change and human activity suggest a likelihood of increased contamination levels with time. Secondly, the chances of misreporting were imminent considering that symptoms of aflatoxicosis, for example, jaundice, oedema, vomiting, severe liver damage and kidney failure (Mwanda *et al.,* 2005; Sayo, 2015; Xu *et al.,* 2018), are similar to those of other illnesses. Thirdly, unless the cases were large enough to be classified as an epidemic, misdiagnosis and underreporting would have likely occurred and isolated cases may have passed unaccounted for.

## Mitigation

Due to the numerous lives lost and high level of exposure to aflatoxins in a majority of the Kenyan population, the health incentive has been a key driver to finding solutions to the aflatoxins problem in the country. Heightened public and private sector participation in mitigation measures have been observed. We describe public and private sector driven initiatives in Kenya dedicated to addressing the aflatoxins problem in the past and present. This section transitions into the next that addresses knowledge gaps worth considering while designing future management efforts.

### The aflatoxins task force of the Kenya government

The aflatoxins task force is an interdepartmental/inter-ministerial team instituted to spearhead surveillance of aflatoxins in maize as well as advise the government on looming outbreaks and containment measures. Its formation was catalysed by the acute aflatoxicosis outbreak that occurred in 2004 that recorded over 125 fatalities and reported several other people with illnesses. It is chaired by the Agriculture Secretary in the Ministry of Agriculture and Irrigation (over the years and across regimes, the agriculture and livestock docket has been domiciled in a ministry that has borne different names) and has representatives from the agriculture and livestock ministry, Kenya Agricultural and Livestock Research Organization (KALRO), Ministry of Health, Kenya Bureau of Standards (KEBS), Kenya Plant Health Inspectorate Services (KEPHIS) and public universities. The task force has since generated reports documenting causative factors for the 2004-2005 aflatoxicosis outbreak and attributed the outbreak to delayed harvesting, poor storage practices that led to excessive moisture in the grain and low awareness about aflatoxins. The functions of the task force are, however, currently being replicated by its member institutions, as the awareness about the dangers of aflatoxins continues to amplify (K. Mutambuki, 2018, personal communication).

### National Food Safety Coordinating Committee

The National Food Safety Coordinating Committee (NFSCC) was constituted in February 2006 with broad consultations among stakeholders. The Secretariat is the Ministry of Health, while the chairmanship lies with the State Department of Agriculture – Ministry of Agriculture and Irrigation. Its membership is drawn from ministries/state departments/Semi-Autonomous Government Agencies (SAGA) of health, agriculture, livestock, fisheries and trade; academia; and the Council of Governors of the government of Kenya. The Food and Agriculture Organization of the United Nations (FAO), World Health Organization (WHO), World Food Program (WFP), Kenya Association of Manufacturers (KAM) and Cereal Millers Association (CMA) are also co-opted members. Its mission is to protect consumers’ health by ensuring that food produced, distributed, marketed, and consumed meets required standards of food safety. Among its roles are to coordinate formulation of food safety policies and supportive legal framework, harmonise and coordinate the implementation of food control activities including food and feed analysis, inspection, enforce and coordinate food safety information, education and communication (IEC) and monitor and evaluate food safety programs.

The committee is involved in annual testing for contamination by aflatoxins in maize, during and after harvest. The exercise is led by the State Department of Agriculture/NCPB/NFSCC Secretariat. Contaminated maize is subsequently withdrawn. Together with FAO, the NFSCC undertook a needs assessment to: (1) identify stakeholders in the maize value chain and their role and responsibility in the prevention, management and control of aflatoxins contamination; (2) identify and assess the gaps and needs of various stakeholders in their role and responsibility in prevention, management and control of aflatoxin contamination; and (3) identify and recommend opportunities for aflatoxin prevention and control among various stakeholders. Many recommendations from the needs assessment are currently being implemented and include awareness raising, education for agricultural officers and public health officers (the NFSCC secretariat together with FAO trained selected county officers from the county departments of agriculture and health as trainers of farmers – ToF – on aflatoxins prevention and management in 17 out of the 47 counties in Kenya. The 17 counties where farmers were trained included: Meru, Tharaka Nithi, Embu, Kitui, Machakos, Makueni, Laikipia, Kirinyaga, Murang’a, Tana River, Kilifi, Mombasa, Kwale, Taita Taveta, Nandi, Uasin Gishu and Trans Nzoia counties) as well as developing capacity for low-cost testing for aflatoxins.

In their individual capacity, member institutions of the NFSCC have their own aflatoxins mitigation initiatives. For example, the government has initiated the Warehouse Receipting System, in collaboration with the NCPB, and committed to drying and storage of farmers’ grain at a fee. In addition, it has initiated efforts of purchasing mobile driers for installing at various points in the country. Education campaigns are an on-going process in the ministry, with extension officers carrying out education programs just before the maize season’s harvest. The ministry of health is tasked to coordinate aflatoxins analyses of samples of maize collected from various parts of the country, particularly from highly prone regions (R. Kilonzo, NFSCC sub-committee briefing meeting on aflatoxins at the Intercontinental Hotel, Nairobi on 1 March, 2018, unpublished data). In 2018, the Partnership for Aflatoxin Control in Kenya (PACK) was conceived during the implementation of a Technical Cooperation Program (TCP) between the Government of Kenya through the NFSCC meeting (NFSCC – 2017) and FAO (FAO/NFSCC – TCP/KEN/3402) on prevention and management of aflatoxins along the maize value chain. PACK is meant to serve as a knowledge and information management platform accessible to all stakeholders. As of this writing, the platform is yet to be formalised, but incountry stakeholder consultations are currently underway (R. Kilonzo, NFSCC sub-committee briefing meeting on aflatoxins at the Intercontinental Hotel, Nairobi on 1 March, 2018, unpublished data).

### Regulatory compliance through national standards and regulatory bodies

Following the death of numerous dogs after eating commercial dog feed in the 1980s, the standards and regulatory agency, KEBS, drafted a standard for dog feeds in 1985. Standards for maize grain and other food grains and products existed before this date and had been set at 20 μg/kg. This standard was thereafter revised by KEBS to 10 μg/kg for total aflatoxins and 5 μg/kg for AFB_1_ in 2007 (KEBS, 2007), after conducting a risk assessment based on dietary exposure, frequency of consumption of highly prone food items particularly maize, methodologies available for aflatoxins testing, and available literature on prevalence and exposure. To date, there are over 30 standards related to aflatoxins control, including management of associated parameters, such as moisture and condition of grain and feed that cover several commodities in Kenya. These include KS CAC/RCP 45:1997, KS EAS 57:2000, KS CAC/RCP 59:2010, KS CONSTAN193:2015 and KS EAS 2:2017, among others (KEBS, 2018b). Enforcement of standards has been a major challenge as regulatory entities have focused on the organised formal sector, leaving out the informal sector through which over 90% (Amenya, 2007) of the food supply in Kenya is transacted. Amongst those served by the unregulated informal sector include upper middle, lower middle, low-income populations and the very poor, who subsequently become exposed to aflatoxins (Leroy *et al.*, 2015). In a predominantly unregulated and informal sector environment, the net benefit of revising aflatoxins standards downwards requires quantification (Sirma *et al.*, 2018).

### Collaborations between the Ministry of Agriculture and Irrigation and FAO

Following the acute aflatoxicosis incidence in 2004 that resulted in numerous deaths, FAO supported the Ministries of Agriculture and Health officers to identify causative factors for the outbreak as well as introduce measures to arrest future occurrences. As part of the response, extensive awareness raising about aflatoxins was done mainly through public and private sector stakeholders consultations both at the national and county levels. The county officers who participated in awareness raising were encouraged to disseminate the same information through *barazas* and any other relevant networks. *Barazas* are public gatherings organised under the provincial administration to deliver messages that are deemed crucial to the public. They are localised and can be organised within the smallest administrative unit of government. As part of the mitigation measures, moisture meters and aflatoxins testing equipment were provided to extension agents to assist farmers in monitoring moisture content in grain and undertake surveillance. Long-term solutions proposed included development of early warning systems, revision of aflatoxins standards to ensure optimal safety for the end consumer while ensuring fair trade; establishment of aflatoxins prevalence data to identify areas requiring immediate intervention; testing/developing aflatoxins management options; continuous monitoring and surveillance programs; capacity building and strengthening aflatoxins-related policy and regulation (S. Kimereh, 2018, personal communication). The structuring of the relationship between the Kenya government’s agriculture and public health departments and FAO presents notable lessons for future initiatives. These lessons include allocation of resources within on-going government programs to ensure continuity of programs after project completion (resources are allocated to augment rather than duplicate extension services); and alignment of its in-country activities with the Kenya government’s agricultural agenda, making implementation impactful.

### The US Centres for Disease Control and Prevention, through the Public Health Department of the Health Ministry

The duo conducted epidemiologic investigations to assess exposure levels and resulting health effects, as well as identify modifiable risk and protective factors for consuming aflatoxins contaminated food. The US Centres for Disease Control and Prevention (CDC) evaluated the performance of rapid screening tests (Y.A. Redwood, unpublished data). Assessing the performance of a rapid screening tool to detect aflatoxins in maize in Eastern Kenya) in rural village settings and the feasibility and cost-effectiveness of sustaining local food screening programs in rural, vulnerable populations. In an intervention study, it was demonstrated that inclusion of calcium silicate 100 (ACCS100), a calcium montmorillonite clay in the human diet reduced the bioavailability of aflatoxins. Although ACCS100 was effective, acceptable, and palatable, the authors suggested further evaluation of ACCS100 among people in aflatoxins-prone areas and to determine if ACCS100 remains effective at the levels of aflatoxins exposure that induce aflatoxins poisoning (Awuor *et al.*, 2017).

### Public sector and the Consultative Group on International Agricultural Research Centres

The potential merit of utilising indigenous atoxigenic *A. flavus* strains to manage aflatoxins was suggested by Probst *et al.* (2011). The International Institute of Tropical Agriculture (IITA), United States Department of Agriculture – Agricultural Research Service (USDA-ARS), KALRO, and the National Irrigation Board embarked on on-station and on-farm trials that led to the development of a biocontrol product, Aflasafe KE01™. KALRO is the registrant of the product. The product comprises of four indigenous atoxigenic (incapable of producing aflatoxins) strains of *A. flavus* found across several regions in the country (Bandyopadhyay *et al.*, 2016). The Kenyan government has used the product for aflatoxins mitigation in highly aflatoxins-prone counties that include Makueni, Machakos, Kitui, Embu, Tharaka-Nithi, Meru, Kilifi and Tana River. Initially, nearly 240 tons of Aflasafe KE01 were imported from Nigeria, but a manufacturing plant was established in KALRO in 2017 to cater for the local demand at an affordable cost and to build local capacity to manufacture Aflasafe KE01. Additionally, together with the Ministry of Agriculture and NIB, IITA has trained more than 7,000 farmers in the management of aflatoxins.

IITA also partnered with the East African Community (EAC) states and the EAC secretariat to develop technical papers on various topics about aflatoxins. Some of the topics of these papers are pre- and post-harvest management, food and feed standards, biological control as an aflatoxins management option, aflatoxins and human health, aflatoxins and hepatitis A and B, aflatoxins and the first 1000 days (focussing on the formative years of a child since birth, when nutritional health challenges can begin), and communication. The papers served as the foundation for the development of policy briefs to enhance aflatoxins mitigation in the region (East African Community, 2018b). In 2018, the EAC council of ministers approved the aflatoxins prevention and control strategy 2017-2022, a product developed by the elaborate policy process. The action plan and results framework of the strategy lays out issues related to the implementation of the strategy at the regional and national levels (D. Wafula, Report of the 36^th^ meeting of the council of ministers on 20 February, 2018, Kampala, Uganda. Ref-EAC/CM/36/2017, unpublished data).

The Consultative Group on International Agricultural Research (CGIAR) centres have also been instrumental in building local capacity for aflatoxins mitigation through short term and long term formal trainings in partnership with local and international institutions of learning and within training facilities within the CGIAR centres. For example, IITA has partnered with KALRO to advance capacity building efforts through the East Africa regional mycotoxin research facility within KALRO, while the International Livestock Research Institute (ILRI) has played a similar role through the Biosciences Eastern and Central Africa (BECA) platform, where a mycotoxin training and diagnostics platform exists. ILRI, together with national partners, has also played a significant role to elucidate the role of aflatoxins in the livestock value chain. The International Maize and Wheat Improvement Centre (CIMMYT) has partnered with the national programs to advance breeding as a long-term measure for pre-harvest aflatoxins mitigation, while the International Food Policy Research Institute (IFPRI) has led efforts to test ready to scale technologies for mitigation of aflatoxins particularly for the smallholder farmer and generate human exposure information. The International Crops Research Institute for the Semi-Arid Tropics (ICRISAT) in Kenya has focused on establishing the prevalence of aflatoxins in peanuts at the household and market levels, and education on pre-harvest and postharvest mitigation measures for contamination by aflatoxins in peanuts (Grace *et al.*, 2015).

### Other public-private partnership initiatives

The Aflatoxin Proficiency Testing and Control in Africa (APTECA) program is being piloted in various African countries, including Kenya, where CMA, BECA platform, and several government departments have partnered to advance a quality systems approach for managing aflatoxins. The Cereal Growers Association (CGA) continuously partners with private and public-sector players to educate farmers on aflatoxins mitigation and test ready to scale technologies in their mandate maize growing regions. The Mexican government recently partnered with the public sector including KALRO and the local universities, as well as private sector to advance nixtamalization (alkaline cooking) as a postharvest intervention to manage aflatoxins in maize grain.

The ACDI-VOCA, an international not-for-profit, nong-overnmental organisation has assisted commercial maize farmers in improving the quality of their grain through sound postharvest practices. ACDI-VOCA has also partnered with other private sector players, government, and CGIAR centres to raise awareness about aflatoxins among farmers and extension officers through dissemination workshops. The recently concluded AflaSTOP project, a collaboration between ACDI-VOCA, Agribusiness Systems International, Meridian Institute and several public-sector institutions, has generated important information on comparative cost-effectiveness of existing hermetic storage technologies (Walker *et al.*, 2018) and commercialisation feasibility of the EasyDry M500 portable dryer.

Several cereal value chains and farmers have benefited from the Purchase for Progress (PFP) process set up by the WFP that ensures that the international humanitarian organisation purchases safe and quality grain from farmers after independent testing (Meaux *et al.*, 2013). In some instances, there have been joint public-sector initiatives, such as the Safe Dairy Project, an effort of the University of Nairobi, Agrifood Research Finland (MTT), Finnish Food Safety Authority (EVIRA), KALRO and Egerton University in Kenya. The Safe Dairy project had a major thrust on strengthening the dairy sector through better animal health by improving dairy management practices that include safe feed (E. Kang’ethe, 2014, personal communication).

The Partnership for Aflatoxin Control in Africa (PACA) has played an important role in the mitigation of aflatoxins in Africa. Through the leadership of the AU and supported by a steering committee that has representation across sectors and disciplines, it has provided a platform for engaging and deliberating solutions to aflatoxins in the continent to safeguard consumer health and facilitate trade (PACA, 2018). Kenya has been a beneficiary of some of the regional initiatives that have been supported through PACA, including infrastructure development for advancing technologies such as the use of biological control for mitigation of aflatoxins and capacity building through infrastructural support.

The initiatives described above demonstrate the effectiveness of public-private partnerships that optimize on each partner’s complementary strengths such as existing and trusted in-country mechanisms; infrastructure and regulation within the public sector to support information and technology dissemination; strong technical backstopping from private sector players; pooling of resources from partners; mainstreaming proposed initiatives within government structures; and utilizing public and privates sector platforms for information dissemination.

## Economic drivers for mitigation of aflatoxins

The motivation to invest in management efforts is driven by aspects of the intervention that affect perception, uptake, buy-in and scaling. A major push for seeking solutions to address contamination by aflatoxins in Kenyan produce is the historical negative impacts on the health of the population. Wu (2010) quantified health losses associated with contaminated produce. The cost of treating such health conditions is a burden, to the affected families and the government health institutions providing treatment services. Moreover, the cost of technology directly affects adoption and upscaling efforts. Costs must make economic sense to the end user and the supplier of the technology. Several technologies have been advanced for use at smallholder level, but few studies have determined the economic incentives to promote uptake. Household income is another factor that determines the success of uptake of a technology. The majority (80%) of farmers in Kenya operate smallholder enterprises that are resource deficient, and in the absence of adequate knowledge, are unlikely to prioritise aflatoxins mitigation measures. In a cross-sectional study undertaken by Leroy *et al.* (2015), high aflatoxins exposure levels were strongly associated with poverty while household expenditure, food security, use of organic fertilisers and pesticides and owning more land were associated with less exposure.

Another constraint to adoption of aflatoxins interventions is the lack of an economic incentive for aflatoxins standard-compliant food, due to lack of market differentiation. Most markets in sub-Saharan Africa do not differentiate aflatoxins-compliant products from unsafe ones. A well-informed consumer-population would be able to increase demand for aflatoxins-compliant products by asking for the same. For information dissemination initiatives to be successful, the content and strategy to communicate about aflatoxins must be well thought out. Beyond alerting on the acute dangers of aflatoxins, messaging needs to underscore chronic exposure and practical solutions to targeted audiences; dissemination must provide a message of hope rather than reprimand.

The intended scope of use of aflatoxins interventions can also affect uptake. Some aflatoxins mitigation technologies are better suited for largescale operations, e.g. bulk grain driers, grain silos and cocoons, use of aircrafts for pesticide application, compared to smallholder farming operations, due to cost, efficiency, energy, and scale considerations. Technologies for large-scale operations such as bulk drying and storage facilities can, however, be beneficial for smallholder farmers when their grains are aggregated, handled and marketed by farm-based business enterprises.

## Knowledge gaps and prioritisation of research efforts

Heightened awareness of dangers linked to aflatoxins has resulted in increased studies to generate further information on prevalence (including of other types of mycotoxins), and test and/or develop mitigation measures. Many mycotoxins co-occur, as their causal agents grow on similar substrates and thrive in similar environmental and climatic conditions. On the contrary, sparse information is available on human exposure and associated factors, such as contamination levels, sex, age, dietary habits, occupation and immunity (Mehan *et al.,* 1991). Moreover, there is barely any evidence on the economic impact from reduced trade, increased cost of managing aflatoxins related illnesses and death, which leads to loss of livelihoods, and the existence of alternative uncensored markets for contaminated produce in the country. Rigorous tools to measure economic losses are, therefore, required to establish the impact of aflatoxins and effectiveness of interventions to promote strategies that demonstrate optimal impact at a reasonable cost.

The review also shows a heavy bias of data collected from the eastern region of Kenya, compared to other parts of the country, elicited by the acute aflatoxicosis cases reported from the region as well as the presence of pre-disposing factors. The review, however, shows that contamination and exposure occur in other regions of Kenya, warranting the need to extend in depth aflatoxins studies and resulting impacts to these regions.

A major obstacle in designing research plans for effective data generation is the absence of tested and approved sampling strategies that suit the crop and animal production systems of smallholder farmers in sub-Saharan Africa. Past and on-going research in the country has had to customise sampling protocols suited for large lots. Some widely-used sampling methods include those developed by FAO and EU expert groups (FAO, 1993; EC, 2006). Partnering with member state institutions and experts, Common Market for Eastern and Southern Africa (COMESA) recently developed sampling methods to enhance trade. Many of these methods still have shortcomings in the context of smallholder farming systems due to the small quantities of grain available in farmers’ fields and stores and limited choice in sample collection and aggregation tools. Berry and Day (1973) studied suitable ways of analysing skewed data. The authors used data on levels of aflatoxins in foods collected in the Murang’a District of Kenya, to investigate the aetiology of liver cancer (Peers and Linsell, 1973). Out of 2,432 samples collected, only 124 had detectable (>1 μg/kg) levels of aflatoxins. The distribution of the measurable values was highly skewed. The authors found that the goodness of fit of the gamma distribution best described such data. Their findings have been a basis for analysing similar data in many instances. Aspects to be considered in future efforts to design sampling strategies for products of similar nature and in smallholder production systems include lot size, sample collection tools and processes.

The use of clays in reducing human exposure to aflatoxins has been tested in Kenya (Obura *et al.*, 2017). Alkaline treatments of maize and groundnut produce have also been tested (Mutungi *et al.,* 2008) with promising outcome in aflatoxins reduction. The practicability of using this strategy as a household intervention measure needs to be explored further, including the cost-benefit analysis, organoleptic properties and ethical considerations.

The gap between agriculture, health and nutrition sectors’ engagement is a cause for the heavy disease burden resulting from agriculture (Grace *et al.*, 2015). Reducing these gaps must receive high priority. Research interventions must also accommodate the informal grain value chains, owing to the large grain volumes (over 90%) transacted through such avenues. Already, some work has been done on quantification of the impacts of aflatoxins on various facets, e.g. trade, health (Kang’ethe *et al.,* 2017; Okoth and Ohingo, 2004; Wu, 2010), but many more data need to be gathered at the national level to provide impetus for use of various mitigation measures. For example, in as much as several studies have associated aflatoxins exposure with stunting, the extent to which stunting roots from exposure to aflatoxins needs to be quantified through a cause-effect approach (Leroy, 2013).

DeGroote *et al.* (2016) demonstrated a willingness to pay for maize tested for aflatoxins and labelled by rural consumers in maize producing regions of Kenya, particularly those with adequate knowledge and from heavily affected areas in Kenya. Willingness to pay needs to be determined for several other technologies available for upscaling and out scaling to gauge their acceptability and reasons for resultant adoption levels.

Several studies have shown the effectiveness of sorting in the reduction of aflatoxins and other mycotoxins at various levels (Afolabi *et al.*, 2007; Hell and Mutegi, 2011; Xu *et al.*, 2017). Improvements in reducing subjectivity in the sorting processes have been explored (Stasiewicz *et al.*, 2017) and require to be advanced to improve throughput and quantification of aflatoxins reduction levels resulting from sorting.

Potential lies in the use of genetic tools for management of aflatoxins, for example through gene silencing (Sharma *et al.*, 2018). However, a major regulatory impediment in the country is the restricted commercialisation of genetically modified produce even though its application is approved at the research level. Additionally, an understanding of the population structure of aflatoxins producing fungi in Kenya has helped in targeting interventions (Probst *et al.*, 2014). Such studies must, therefore, continue as they help in understanding the progression/reduction of the problem, and factors associated with such trends. The extent of climate change on the growth and proliferation of the *A. flavus* community and potential to produce aflatoxins cannot be ignored. An integrated approach for aflatoxins mitigation measures has been recommended at various levels that take into consideration the volatility of the impacts of climate change (Bandyopadhyay *et al.*, 2016; Wambui *et al.*, 2016b). Furthermore, investigations on the effects of agronomic traits and soil health on aflatoxins that have been studied (Mutiga *et al.,* 2017) need to be advanced to a conclusive outcome.

Some technologies that have demonstrated high efficacy have not moved beyond the laboratory due to the impracticability of scaling them, despite their effectiveness in reducing levels of aflatoxins. Some of these are chemical treatments whose safety has not been researched while for others, the cost of scaling has not been justified. For example, the potential for use of food grade super absorbent polymers (SAP) in the reduction of aflatoxins by targeting moisture reduction has been explored (Mbuge *et al.*, 2016) and requires further investigation on safety, cost-effectiveness and scaling.

## Conclusions and recommendations

It is evident that little research was undertaken in Kenya before 2004, either to establish prevalence or introduce management options. Indeed, investments in aflatoxins research gained impetus in the later years of the first decade of the 21^st^ century, as evidenced by the large amount of research outputs documented during this phase, particularly prevalence data. Before then, grey literature and media content served as important sources of information on aflatoxins in the country.

The high levels of aflatoxins in food and feed commodities including in Kenya’s key staple starch source, maize, is conclusively evident. We deduce that prevalence data generated so far in Kenya is adequate to ignite a shift of resources, towards management. Public sector players that are important in implementing management programs in the country include SAGAs in agriculture, livestock, fisheries, health and trade, extension services in agriculture and health, the office of the president, universities and the NFSCC. Their roles straddle developing aflatoxins related standards and ensuring that they are implemented; monitoring, surveillance and seizure of contaminated grain; testing of grain; capacity building (infrastructure, short- and long-term training), policy development and implementation, emergency responses, research, planning and resource support to encourage sustained efforts.

The private sector is critical in complementing public sector efforts and providing human capacity and technologies through their open access fora. They contribute to institutionalising appropriate aflatoxins management systems to ensure that aflatoxins remain under control in the formal food and feed sectors. Joint efforts in advancing the management of aflatoxins must therefore be encouraged. Non-governmental public institutions such as the CGIAR centres have been useful in augmenting government efforts particularly in technology development and dissemination, while development partners such as United States Agency for International Development (government), Bill and Melinda Gates Foundation (private), United Nations Food and Agriculture Organization (intergovernmental), the European Union (intergovernmental), African Union (intergovernmental), continue to supplement state resources to support various interventions. Regional bodies such as COMESA, EAC, and the PACA platform serve a multifaceted role that includes influencing member states to actively participate in the development and implementation of aflatoxin interventions; developing aflatoxin related policy and capacity building. Such support notwithstanding, the State must take a lead in setting priorities in mitigation of aflatoxins to ensure sustainability. Efforts must, therefore, be entrenched in the government’s agricultural strategic agenda.

To effectively implement management of aflatoxins, capacity development in the form of human resource base and infrastructure is necessary. The human capacity to address various facets of aflatoxins mitigation is still low. Up-to-date research facilities for mycotoxin research, for food commodities and human and animal exposure, is required particularly in public institutions. The relevance of social learning and networks in promoting aflatoxins mitigation efforts amongst smallholder farmers need to be considered (Ngotho, 2017). Gender roles are an important consideration in making decisions on food handling at the household level (Kiama *et al.,* 2016). Subsequently, they form an important consideration in recommending household measures for mitigation of aflatoxins and should not be ignored.

Diagnostic tools and testing equipment must be properly targeted. Their application needs to ruminate on cost, availability, rapidity in decision-making process on the testing outcome, the scale of use, whether the grain is for home or market consumption, as well as the capacity of the end user to competently and appropriately use the testing methods. Alongside, sustained public awareness is necessary to develop a population that is conscious of the benefits of consuming safe food and consequently demand for it.

Finally, government and private sector can play a crucial role in strengthening policies that impact on food safety, as well as support risk assessment initiatives to ensure that well thought out standards for mycotoxins are in place. Scarce resources available to advance management efforts must be utilised well by proper targeting and ensuring that duplication of efforts is minimised. Unlike in several other countries where trade primarily drives the impacts of aflatoxins and attempts to get solutions, Kenya has suffered immensely from health impacts to the extent that the government declared it a national disaster. The incentive to find lasting solutions to the problem in the country is, therefore, health and trade-based.
